# Association of lung diffusion capacity with cardiac remodeling and risk of heart failure: The Framingham heart study

**DOI:** 10.1371/journal.pone.0246355

**Published:** 2021-02-16

**Authors:** Ibrahim Musa Yola, Albin Oh, Gary F. Mitchell, George O’Connor, Susan Cheng, Ramachandran S. Vasan, Vanessa Xanthakis

**Affiliations:** 1 Section of Preventive Medicine and Epidemiology, Department of Medicine, Boston University School of Medicine, Boston, MA, United States of America; 2 Department of Medicine, Boston Medical Center, Boston, MA, United States of America; 3 Cardiovascular Engineering, Inc., Norwood, MA, United States of America; 4 Department of Medicine, Pulmonary Center, Boston Medical Center, Boston University, Boston, MA, United States of America; 5 Smidt Heart Institute, Cedars-Sinai Medical Center, Los Angeles, CA, United States of America; 6 National Heart, Lung, and Blood Institute, Framingham Heart Study, Framingham, MA, United States of America; 7 Department of Epidemiology, Boston University School of Public Health, Boston, MA, United States of America; 8 Department of Biostatistics, Boston University School of Public Health, Boston, MA, United States of America; Scuola Superiore Sant’Anna, ITALY

## Abstract

**Background:**

Lung function abnormalities are ubiquitous in heart failure (HF). It is unclear, however, if abnormal lung diffusion capacity is associated with cardiac remodeling and antedates HF. We hypothesized that lower lung diffusion capacity for carbon monoxide (DLCO) is associated with worse left ventricular (LV) systolic and diastolic function cross-sectionally, and with higher risk of HF prospectively.

**Methods:**

We evaluated 2423 Framingham Study participants (mean age 66 years, 55% women) free of HF who underwent routine echocardiography and pulmonary function tests. We used multivariable regression models to relate DLCO, forced vital capacity (FVC), and forced expiratory volume in 1 second (FEV1) to left ventricular ejection fraction (LVEF), left atrial (LA) emptying fraction (LAEF), E/e’, E/A, LV mass, and LA diameter (LAD). Multivariable-adjusted Cox proportional hazards regression was used to relate DLCO, FEV1, and FVC to incident HF.

**Results:**

In multivariable-adjusted cross-sectional analyses, DLCO, FEV1, and FVC (dependent variables) were associated positively with LVEF (β_DLCO_ = 0.208, β_FEV1_ = 0.021, and β_FVC_ = 0.025 per 5% increment in LVEF; p<0.005 for all), and LAEF (β_DLCO_ = 0.707, β_FEV1_ = 0.058 and β_FVC_ = 0.058 per 5% increment in LAEF; p<0.002 for all). DLCO and FVC were inversely related to E/A (β_DLCO_ = -0.289, β_FVC_ = -0.047 per SD increment in E/A; p<0.001 for all). Additionally, DLCO, FEV1 and FVC were inversely related to HF risk (108 events, median follow-up 9.7 years; multivariable-adjusted hazard ratios per SD increment 0.90, 95% CI 0.86–0.95; 0.42, 95% CI 0.28–0.65, and 0.51, 95% CI 0.36–0.73, respectively). These results remained robust in analyses restricted to non-smokers.

**Conclusions:**

Our large community-based observations are consistent with the concept that lower lung diffusion capacity and expiratory flow rates are associated with cardiac remodeling and may antedate HF. Additional studies are needed to confirm our findings and to evaluate the prognostic utility of pulmonary function testing for predicting HF.

## Introduction

Heart failure (HF) is a condition that affects 6.5 million Americans, and its prevalence is increasing [1]. Once patients are symptomatic with HF, many organ systems are affected [2]. The mechanisms of lung dysfunction in patients with HF have been well described [2–5]. However, less is known about pulmonary function alterations in patients with asymptomatic cardiac dysfunction and it is unclear if such alterations may presage overt HF.

Chronic Obstructive Pulmonary Disease (COPD) is a known risk factor for HF (especially right-sided heart failure) [6, 7]. Even without a clinical diagnosis of pulmonary disease, low FEV1 and FVC are associated with an increased risk of HF [5, 6]. It has been proposed that systemic inflammation, oxidative stress, and changes in intrathoracic pressure due to lung dysfunction may increase the risk of HF in individuals with low FEV1 and FVC [2, 5, 8]. However, prior studies that assessed the relations between baseline FEV1 and FVC and HF risk did not have baseline echocardiograms to evaluate if asymptomatic cardiac dysfunction was already present and served as a confounder or mediator of the observed associations noted above [5, 6].

Prior community-based studies have shown that asymptomatic LV diastolic and systolic dysfunction are prevalent in the community and that both significantly increase the risk of developing HF [7, 9]. On echocardiography, LV systolic function can readily be assessed with left ventricular ejection fraction (LVEF). The ratio of early mitral inflow velocity to mitral annular early diastolic tissue velocity (E/e’) is used often as a marker of LV filling pressure [10]. Higher LV mass increases the risk of both LV systolic and diastolic dysfunction as well as increased left atrial (LA) size [11]. In patients with LV dysfunction, the LA is subject to increased volume and pressure overload that can result in LA remodeling, enlarged LA size and dysfunction, ultimately decreased LA emptying fraction [12–14].

Knowing that asymptomatic LV dysfunction often precedes clinical HF [9], we hypothesized that the subtle lung function abnormalities may also be associated with LV systolic, LV diastolic, and LA dysfunction in asymptomatic individuals. More specifically, using longitudinally acquired data from a community-based population and a retrospective design, we hypothesized that in people without HF, DLCO, FEV1 and FVC are associated with indices of LV and left atrial remodeling. Prospectively, we hypothesized that lower DLCO, FEV1 and FVC are associated with increased risk of HF and of its subtypes, HF with preserved ejection fraction (HFpEF) and HF with reduced ejection fraction (HFrEF).

## Methods

### Study sample

This investigation included Framingham Offspring Study participants who attended their eighth quadrennial examination cycle when they underwent routine echocardiography and pulmonary function tests (n = 2423). For the prospective component relating pulmonary function indices to incident HF, participants with prevalent HF were excluded (n = 45), resulting in a sample of 2378 eligible for the longitudinal analyses. Approval from the Boston University Medical Center Institutional Review Board was obtained for the study protocol and all study participants provided written informed consent.

### Pulmonary and echocardiographic measurements

For the pulmonary function tests (PFT), the variables FEV1, FVC and DLCO were assessed using spirometry based on a standardized protocol described previously [15]. For FEV1 and FVC, spirometry was performed three times with training of participants to exhale as hard and fast as possible. The highest FEV1 and FVC values from the three trials were used. For the DLCO measurements, tracer carbon monoxide was inhaled into the Spirometer with a deep breath (at least 90% of their vital capacity) and participants held their breath for 10–12 seconds. The difference between the inhaled and exhaled carbon monoxide was used to calculate the DLCO. This was done twice at least 4 minutes apart to allow for carbon monoxide wash out. The average of the two values was used to determine the DLCO.

For the echocardiographic measurements, the variables LVEF, LAEF, E/e’, E/A, LAD, and LV mass were assessed using transthoracic two-dimensional echocardiography with pulsed wave Doppler and tissue Doppler imaging based on a standard protocol [16]. LV systolic function was determined by calculating LVEF using the method of de Simone complemented by the visual assessment of LV systolic function [17]. LV diastolic dysfunction was determined by E/e’ (ratio of peak blood flow velocity across the mitral value to peak tissue Doppler movement of the mitral valve annulus in early diastole). We also evaluated the E/A ratio (ratio of peak blood flow velocity from LV relaxation in early diastole [the E wave] to peak blood flow velocity in late diastole caused by atrial contraction [the A wave]) [18, 19]. End diastolic parasternal long axis M-mode measurements were used to calculate LV mass using a validated equation: LV mass (grams) = 0.8 x [1.04 x (LVID+SWT+PWT)^3^−(LVID)^3^]+0.6, using LV internal diameter (LVID), LV septal wall thickness (SWT), and LV posterior wall thickness (PWT) [20]. LAD was determined using M-mode measurements for the end systolic anterior-posterior LA diameter. LA maximum and minimum volumes were determined by averaging values from the area-length method in the 2 and 4 chamber views in order to calculate the LA emptying fraction using [LA_max_−LA_min_]/LA_max_*100 [21–23].

### Covariate definitions

Data were collected during examination cycle 8 (2005–2008) using standardized protocols. Fasting blood samples were collected for blood concentrations of total and high-density lipoprotein cholesterol. All blood samples are stored at - 80°C until assayed [24]. Diabetes was defined as a fasting glucose ≥126mg/dL or use of medications to lower blood glucose. Medication history for prescribed antihypertensive and anti-diabetic agents were self-reported and reviewed by physicians. Participants self-reported if they had smoked in the year before the Heart Study examination to determine current smoking status. An average of two systolic and diastolic blood pressure measurements taken 5 minutes apart on the right arm of the seated participant by a single physician was used for analysis.

### Outcomes of interest

The outcome of interest for this investigation was incidence of HF and its subtypes HF HFpEF and HFrEF. Participants were under surveillance from the time of their eighth examination until the development of a HF event or censoring. A review panel of three physicians adjudicated each HF event. The Framingham criteria were used to define a diagnosis of clinical HF [25]. LVEF at time of diagnosis was used to classify HF as HFpEF (LVEF ≥50%) or HFrEF (LVEF <50%) [17].

### Statistical methods

We used multivariable-adjusted linear regression models to relate FVC, FEV1, and DLCO (dependent variables, separate model for each) to LVEF, LAEF, E/e’, E/A, LAD, and LV mass (independent variables, separate model for each). Models were first adjusted for age, sex, smoking, and height due to their reported relations with FVC, FEV1 and DLCO [26, 27] and further adjusted for weight, history of myocardial infarction (MI), diabetes, total cholesterol/high-density lipoprotein cholesterol (HDL), systolic blood pressure (SBP) and hypertension treatment. A Bonferroni-adjusted p-value of <0.0027 (0.05/18, to account for number of variables tested) was used to account for multiple statistical testing.

Cox proportional hazards regression models were used to assess the relations between pulmonary function test variables (DLCO, FEV1, and FVC; separate model for each) and time to HF after confirming that the assumption of proportionality of hazards was met. Models were adjusted for age, sex, height, weight, smoking, and history of MI, diabetes, total cholesterol/HDL, SBP, and hypertension treatment. Multivariable-adjusted splines were generated to assess for potential nonlinearity of the associations of DLCO, FEV1 and FVC with incidence of HF. Cox proportional hazards regression models for DLCO, FEV1 and FVC were repeated for incidence of HF among non-smokers, as well as for HFrEF and HFpEF separately. Kaplan Meier curves were generated to depict the unadjusted association of tertiles of DLCO, FEV1, and FVC with risk of HF and a log-rank test was evaluated. Analyses were performed in SAS version 9.4. A two-sided p-value of <0.05 was considered statistically significant.

## Results

The study sample consisted of 2423 middle aged adults (66±9 years), with approximately 55% women and an average BMI in the overweight range, as shown in **Table 1**.

**Table 1:** Baseline characteristics of the study sample.

	Men (n = 1080)	Women (n = 1343)
Age, years	66±9	66±9
Body mass index, kg/m^2^	28.9±4.6	27.7±5.8
Height, meters (m)	1.75±0.08	1.60±0.05
Weight, kilogram (kg)	88.45±15.42	71.67±15.88
Current smokers, %	7	9
Systolic blood pressure, mmHg	128±16	127±17
Diastolic blood pressure, mmHg	75±10	73±10
Hypertension medication, %	52	43
Diabetes, %	16	10
Diabetes medication %	11	7
Total cholesterol/HDL	3.7±1.1	3.3±1.0
History of MI, n (%)	23 (2.1)	71 (5.3)
**Echocardiographic features**		
LVEF, %	65±8	69±7
LAEF, %	47±4	48±3
E/e’	6.6±2.1	7.4±2.4
E/A	0.9±0.3	0.9±0.3
LAD, cm	4.2±0.6	3.7±0.5
LV mass, g	201±45	142±32
**Pulmonary Function Test (PFT) features**		
DLCO, mL/min/mmHg	26.4±6.3	18.6±4.0
FEV1, L	3.18±0.71	2.25±0.49
FVC, L	4.41±0.88	3.08±0.60

Data are presented as mean ± standard deviation, unless otherwise noted.

HDL indicates high density lipoprotein cholesterol concentration; LVEF, left ventricular ejection fraction; LAEF, left atrial emptying fraction; E/e’, ratio of early mitral inflow velocity to mitral annular early diastolic tissue velocity; LAD, left atrial diameter; LV, left ventricular; DLCO, lung diffusion capacity for carbon monoxide; FEV1, forced expiratory volume in 1 second; FVC, forced vital capacity.

Adjusting for age, sex, smoking status, and height, DLCO, FEV1, and FVC were positively associated with LAEF, and inversely associated with E/e’. DLCO and FVC were positively associated with LVEF, whereas DLCO was inversely associated with E/A. Furthermore, FEV1 and FVC were inversely associated with LV mass, and FVC was inversely related to LAD (**Table 2**). Upon further adjustment for weight, history of MI, diabetes, total cholesterol/HDL, SBP and hypertension (HTN) treatment, some associations did not retain statistical significance (**Table 2**).

**Table 2:** Cross-sectional associations of pulmonary variables with echocardiographic indices.

	*LAEF*, *per 5%*		*E/e’*, *per SD*		*E/A*, *per SD*		*LAD*, *per 1 cm*		*LVmass indexed by height*		*LVEF*, *per 5%*	
	β (SE)	p-value	β (SE)	p-value	β (SE)	p-value	β (SE)	p-value	β (SE)	p-value	β (SE)	p-value
***DLCO***	0.889 (0.130)	**<0.0001**	-.298 (0.089)	**0.0009**	-0.270 (0.087)	**0.0020**	-0.111 (0.089)	0.2121	0.152 (0.162)	0.3482	0.198 (0.060)	**0.001**
Model 1												
Model 2	0.707 (0.136)	**<0.0001**	-0.272 (0.092)	0.0032	-0.289 (0.087)	**0.0009**	-0.021 (0.096)	0.8289	-0.124 (0.178)	0.4854	0.208 (0.060)	**0.0005**
***FEV1***	0.080 (0.015)	**<0.0001**	-0.041 (0.010)	**<0.0001**	-0.020 (0.010)	0.0445	-.0029 (0.010)	0.0043	-0.050 (0.019)	**0.0070**	0.020 (0.007)	0.0042
Model 1												
Model 2	0.058 (0.015)	**0.0002**	-0.019 (0.010)	0.0723	-0.027 (0.010)	0.0061	0.013 (0.011)	0.2438	-0.040 (0.020)	0.0477	0.021 (0.007)	**0.0022**
***FVC***	0.094 (0.018)	**<0.0001**	-0.071 (0.012)	**<0.0001**	-0.032 (0.012)	0.0070	-0.062 (0.012)	**<0.0001**	-0.094 (0.024)	**<0.0001**	0.025 (0.008)	**0.0022**
Model 1												
Model 2	0.058 (0.0018)	**0.0014**	-0.032 (0.012)	0.0087	-0.047 (0.012)	**< .0001**	0.005 (0.013)	0.6784	-0.065 (0.026)	0.0122	0.025 (0.008)	**0.0016**

Units for DLCO and FEV1 and FVC are mL/min/mmHg and liters, respectively. LVEF, left ventricular ejection fraction; LAEF, left atrial emptying fraction; E/e’, ratio of early mitral inflow velocity to mitral annular early diastolic tissue velocity; LAD, left atrial diameter; LV, left ventricular; DLCO, lung diffusion capacity for carbon monoxide; FEV1, forced expiratory volume in 1^st^ second; FVC, forced vital capacity.

Model 1 adjusted for age, sex, height, smoking.

Model 2 adjusted for age, sex, height, weight, systolic blood pressure, antihypertension medication, history of smoking, diabetes, history of MI, total cholesterol/hdl.

Associations are significant at the Bonferroni-adjusted alpha level (p<0.0027 = 0.05/18).

**Fig 1:**
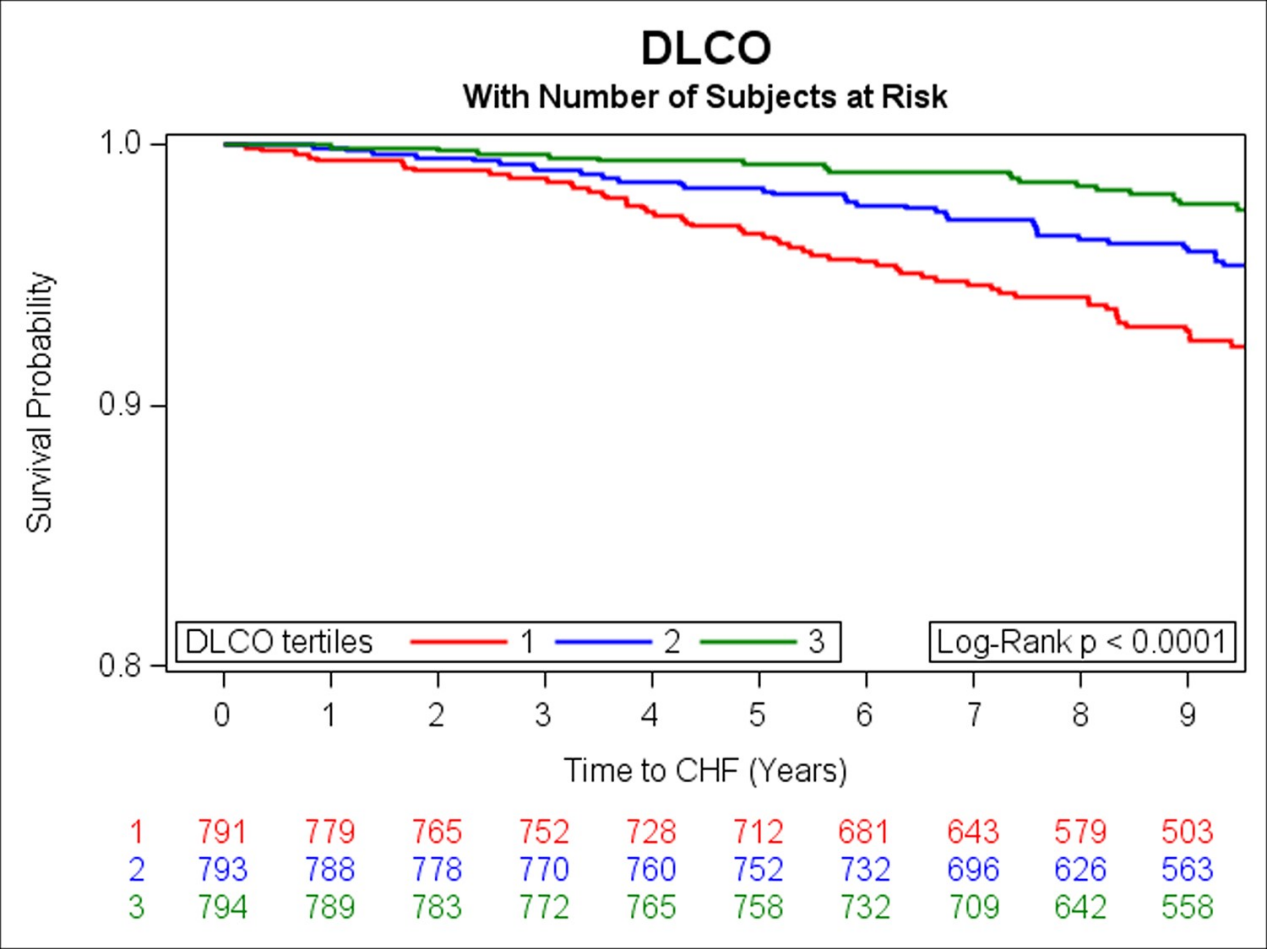
Kaplan-Meier curves depicting the association between tertiles of DLCO and risk of HF (green represents the highest, blue is the middle, and red is the lowest tertile).

**Fig 2:**
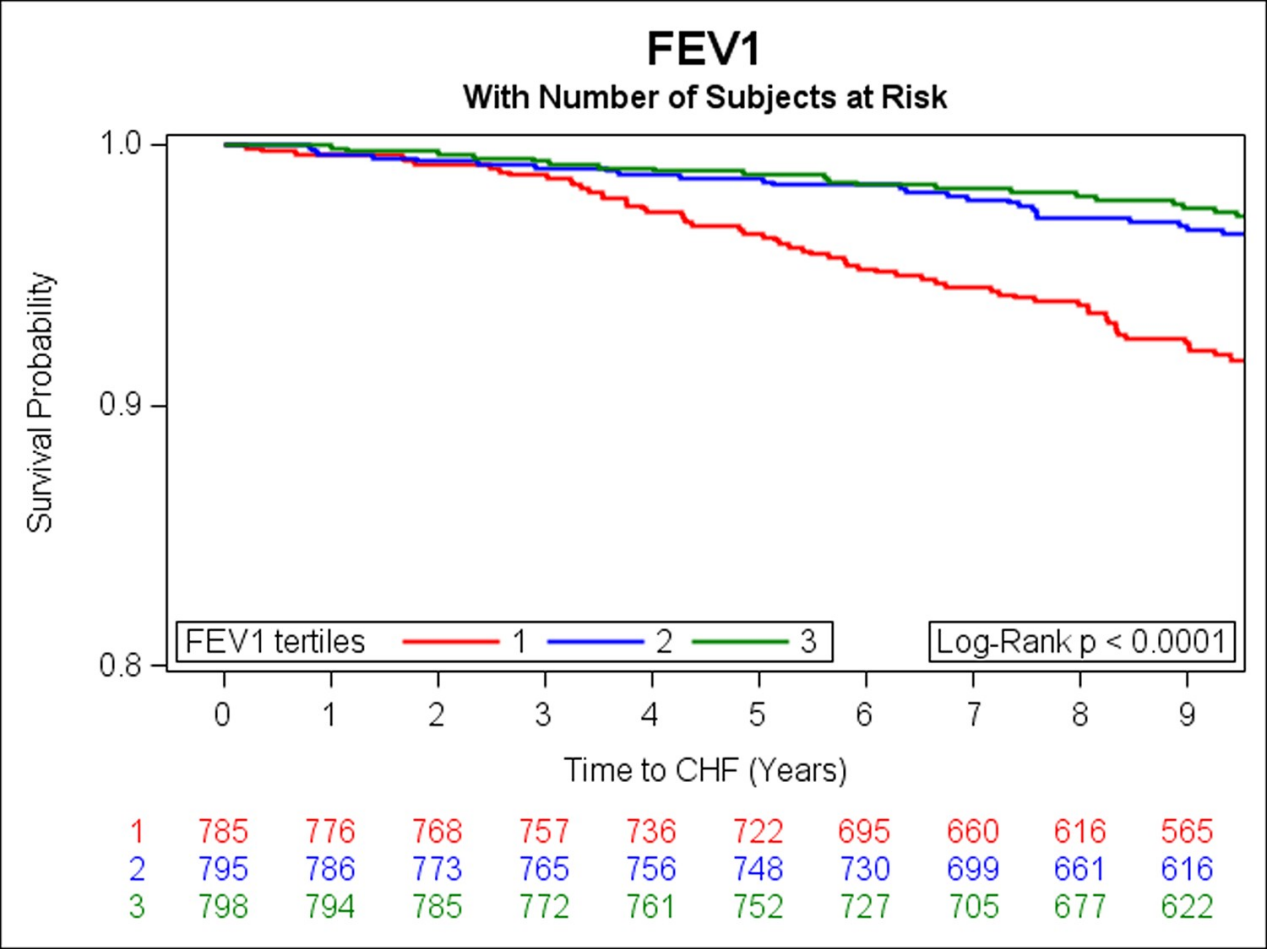
Kaplan-Meier curves depicting the association between tertiles of FEV1 and risk of HF (green represents the highest, blue is the middle, and red is the lowest tertile).

**Fig 3:**
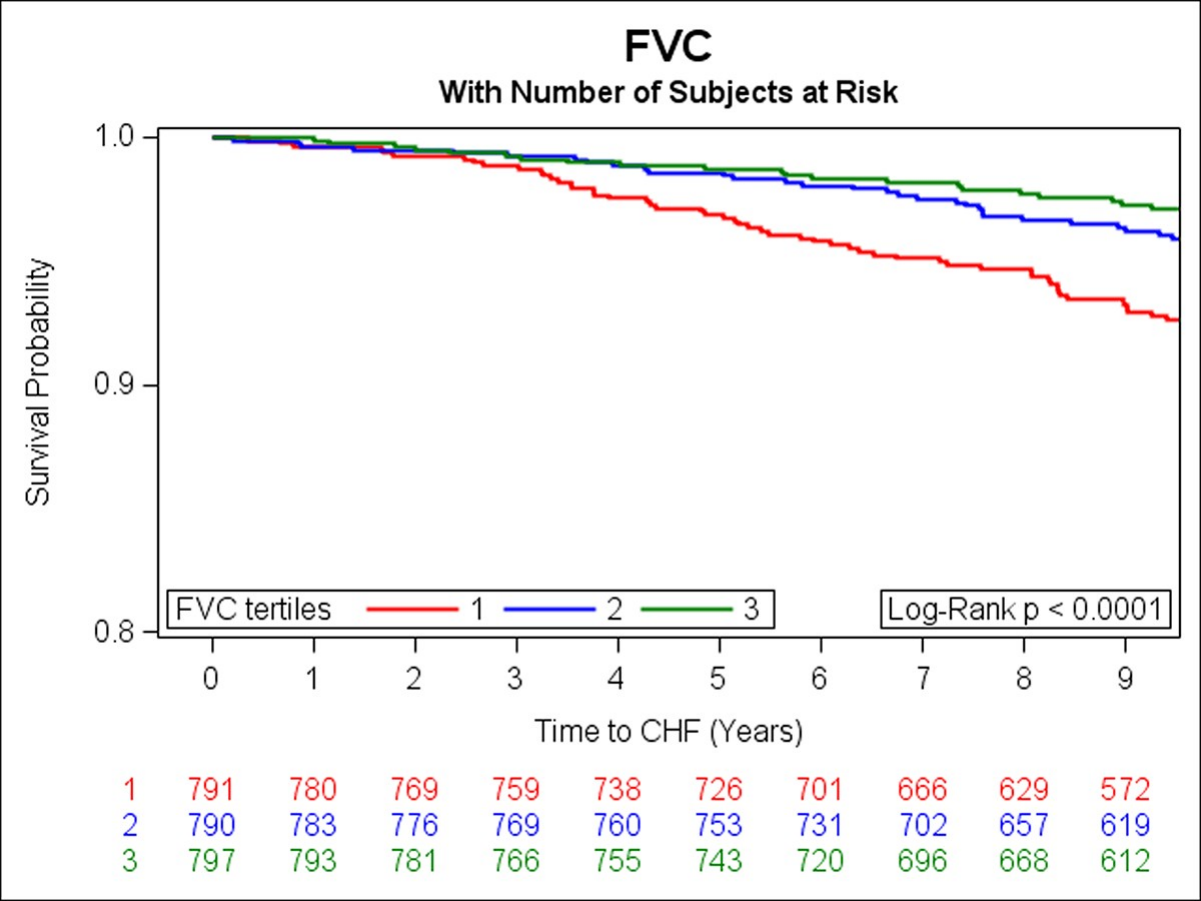
Kaplan-Meier curves depicting the association between tertiles of FVC and risk of HF (green represents the highest, blue is the middle, and red is the lowest tertile).

In the prospective analysis, there were 108 incident HF events over a median follow-up of 9.7 years. Adjusting for age, sex, smoking status, height, history of MI, weight, diabetes, total cholesterol/HDL, systolic blood pressure, and hypertension treatment, DLCO, FEV1 and FVC were inversely associated with risk of HF (**Table 3**). Unadjusted Kaplan Meier (KM) curves depict statistically significant associations of tertiles of DLCO, FEV1, and FVC with risk of HF (**Figs 1–3** respectively). When further adjusting all final Cox models for E/e’, E/A, LVEF, LVM, and LAEF, results were similar (data not shown).

**Table 3:** Associations of DLCO, FEV1 and FVC with incidence of HF.

Model	HF Type	No. of Events/ No. at risk	HR (95% CI)	p-value
All participants				
***DLCO***				
	HF	108/2378	0.90 (0.86–0.95)	**<0.0001**
	HFpEF	46/2339	0.90 (0.83–0.98)	**0.0141**
	HFrEF	39/2332	0.88 (0.81–0.96)	**0.0025**
***FEV1***				
	HF	108/2378	0.42 (0.28–0.65)	**<0.0001**
	HFpEF	46/2339	0.28 (0.14–0.55)	**0.0003**
	HFrEF	39/2332	0.70 (0.36–1.36)	0.2883
***FVC***				
	HF	108/2378	0.51 (0.36–0.73)	**0.0002**
	HFpEF	46/2339	0.34 (0.19–0.61)	**0.0004**
	HFrEF	39/2332	0.71 (0.40–1.25)	0.2282
**Nonsmokers**				
	HF			
***DLCO***	103/2190	0.90 (0.86–0.95)	**0.0001**	
***FEV1***	103/2190	0.39 (0.25–0.61)	**<0.0001**	
***FVC***	103/2190	0.47 (0.32–0.68)	**<0.0001**	

All models were adjusted for age, sex, and current smoking, and height, history of MI, weight, diabetes, total cholesterol/HDL, SBP, and HTN treatment.

**Fig 4:**
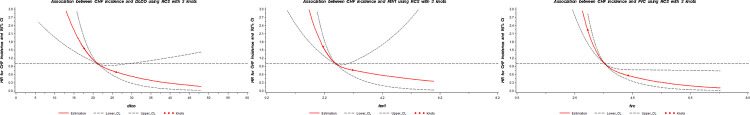
Multivariable adjusted RCS splines assessing for potential nonlinearity of the associations of DLCO, FEV1, and FVC with incidence of HF.

Subgroup analysis was performed among non-smokers, which yielded similar results (**Table 3**). Finally, we observed an inverse relation of DLCO with risk of HFpEF and HFrEF, and of FEV1 and FVC with HFpEF (**Table 3**). Multivariable-adjusted restricted cubic splines did not show any nonlinearity of the observed associations (**Fig 4**).

## Discussion

### Principal findings

First, we observed that DLCO, FEV1 and FVC were positively associated with LAEF and LVEF, while DLCO and FVC were inversely associated with E/A. Second, DLCO, FEV1, and FVC were inversely associated with risk of HF adjusting for potential confounders; these associations remained significant even after limiting analyses to non-smokers. Third, DLCO, FEV1 and FVC were inversely associated with risk of HFpEF, while DLCO was also inversely associated with risk of HFrEF.

### Comparison with the literature

Pulmonary dysfunction, a progressive process in HF, is a common finding and it has been used as a prognostic factor in clinical HF [5, 8, 28–37]. However, its potential relevance as a predictor of preclinical HF has not been explored. We observed that DLCO, FEV1 and FVC were all inversely related to HF risk, similar to prior studies [5, 6]. HF is a progressive disorder, with patients advancing through the stages of HF. Most of what is known about the lungs in HF has been studied in symptomatic patients (ACC/AHA stages C and D HF); it is well recognized that LV systolic and diastolic dysfunction may be associated with increased LV filling pressures, which can cause pulmonary venous congestion [38]. Likewise, LA size is frequently a marker of LV dysfunction and is a risk factor for stroke and mortality [39, 40]. E/e’ and E/A, which are markers of LV diastolic dysfunction have also been used as indirect markers of LV filling pressures [41, 42].

The observations that DLCO, FEV1, and FVC are positively associated with LVEF, LAEF and inversely with E/e’ and E/A suggest that markers of systolic and diastolic cardiac function may be associated with subclinical alterations in pulmonary function. Subclinical cardiac alterations may influence pulmonary vascular hemodynamics, resulting in pulmonary airway and parenchymal remodeling and subsequent alterations in lung function on spirometry.

Prior studies showed that FEV1 and FVC are inversely associated with HF risk and it was proposed that pulmonary dysfunction caused cardiac dysfunction through a variety of mechanisms (systemic inflammation, oxidative stress, changes in intrathoracic pressure) [5, 6]. The foregoing studies were limited in that they did not have baseline echocardiograms for participants, and did not evaluate non-smokers separately.

### Implication of findings

Our investigation suggests that asymptomatic cardiac dysfunction may be associated with lung function alterations, and such alterations may, in turn, influence the risk of developing and manifesting clinical symptoms of HF. These results suggest that cardiac dysfunction may be a precursor of pulmonary abnormalities even in the absence of HF. Current guidelines suggest against the use of pulmonary function testing in asymptomatic patients for COPD, and to our knowledge it has not been evaluated as a screening test for HF [43]. Routine echocardiographic screening for HF has not been recommended because it is impractical and expensive, and requires skilled technicians and interpreters [44]. However, pulmonary function testing is unlikely to prove to be a useful or cost-effective tool for HF screening in the community either. Nonetheless, presence of pulmonary function abnormalities in asymptomatic non-smokers may alert clinicians to the presence of occult lung disease, and also, when the former is excluded, possible subclinical HF or alterations in cardiac function.

### Strengths and limitations

The strengths of our investigation include the large sample size of this community-based cohort and the availability of standardized routine PFT and echocardiograms, as well as the continuous surveillance of individuals for the incidence of HF. Some limitations must be noted. Causality of the observed associations cannot be inferred, given our observational study design. Our sample of predominantly white individuals of European ancestry may limit the generalizability of our findings to other races/ethnicities not studied.

## Conclusions

In our large community-based sample, we observed that spirometric evidence of pulmonary dysfunction might be associated with subclinical cardiac alterations on echocardiogram years before a diagnosis of HF. In addition, lower values of DLCO, FEV1, and FVC may antedate clinical HF. Additional studies are warranted to confirm our findings and to evaluate the utility of pulmonary function testing as a potential tool for risk stratification in Stage B HF.
